# Bromocriptine inhibits proliferation in the endometrium from women with adenomyosis

**DOI:** 10.3389/fendo.2023.1026168

**Published:** 2023-03-09

**Authors:** Yiqun Tang, Sakthivignesh Ponandai-srinivasan, Caroline Frisendahl, Johanna K. Andersson, Dora Pavone, Elizabeth A. Stewart, Parameswaran Grace Luther Lalitkumar, Eberhard Korsching, Nageswara Rao Bogavarappu, Kristina Gemzell-Danielsson

**Affiliations:** ^1^ Division of Neonatology, Obstetrics and Gynecology, Department of Women’s and Children’s Health, Karolinska Institutet, and WHO Collaborating Centre, Karolinska University Hospital, Stockholm, Sweden; ^2^ Division of Reproductive Endocrinology and Infertility, Department of Obstetrics & Gynecology, Mayo Clinic, Rochester, MN, United States; ^3^ Institutet of Bioinformatics, University Hospital of Münster, University of Münster, Münster, Germany

**Keywords:** adenomyosis, bromocriptine, endometrium, proliferation, microRNAs

## Abstract

**Objective:**

Bromocriptine treatment has been shown to reduce menstrual bleeding and pain in women with adenomyosis in a pilot clinical trial. The underlying mechanism contributing to the treatment effect is however unknown. The purpose of this study was to explore the effect of bromocriptine on the proliferation and migration properties of the endometrium in women with adenomyosis, by assessing cellular and molecular changes after six months of vaginal bromocriptine treatment.

**Methods:**

Endometrial specimens were collected during the proliferative phase from women with adenomyosis (n=6) before (baseline) and after six months of treatment with vaginal bromocriptine. Immunohistochemistry was used to determine changes in the protein expression of Ki67 in the endometrium of women with adenomyosis. Primary endometrial stromal cells isolated at baseline were expanded *in vitro* and exposed to different doses of bromocriptine to determine the optimal half-maximum inhibitory concentration (IC50) using CellTiter-Blue^®^ Cell Viability Assay. Cell proliferation was assessed by bromodeoxyuridine ELISA assay and Ki67 gene expression was checked by real-time PCR. The migratory ability of endometrial stromal cells was determined by wound healing and transwell migration assays. Small RNA sequencing was applied on tissues collected from women with adenomyosis before and after bromocriptine treatment to identify differentially expressed microRNAs (miRNAs) after bromocriptine treatment. Bioinformatic methods were used for target gene prediction and the identification of biological pathways by enrichment procedures.

**Results:**

Vaginal bromocriptine treatment reduced the Ki67 protein expression in the endometrium of women with adenomyosis and did not change the prolactin mRNA expression and protein concentration of prolactin in endometrial tissues. Bromocriptine significantly inhibited the proliferative and migrative abilities of endometrial stromal cells derived from women with adenomyosis *in vitro*. Moreover, small RNA sequencing revealed 27 differentially expressed miRNAs between the endometrium of women with adenomyosis before and after six months of vaginal bromocriptine treatment. KEGG pathway analysis on targeted genes of 27 miRNAs showed that several signaling pathways associated with cell proliferation and apoptosis were enriched after bromocriptine treatment.

**Conclusion:**

Bromocriptine treatment exhibits an anti-proliferative effect in the endometrium of women with adenomyosis *in vivo* and *in vitro*. Bromocriptine might inhibit the proliferation of endometrial tissue in adenomyosis in part through the regulation of dysregulated microRNAs and proliferation-associated signaling pathways.

## Introduction

Adenomyosis is a benign uterine disorder, defined by the presence of endometrial glands and stroma infiltration within the myometrium, resulting in uterine enlargement (1). Women with adenomyosis usually present with heavy menstrual bleeding, dysmenorrhea, and chronic pelvic pain (2, 3). Furthermore, accumulating evidence shows that adenomyosis has a negative impact on reproductive outcomes (4). Various treatment options are applied in treating adenomyosis from a complete hysterectomy to non-invasive medications and options in between such as adenomyomectomy, high-intensity focused ultrasound, etc. (5). Current medications include oral contraceptive pills, progestogens, GnRH agonists, and so on. These hormonal treatments typically only relieve symptoms to a variable and unpredictable extent and side effects are usually observed. Novel drugs such as oxytocin antagonists (6), dopamine agonists (7, 8), and anti-platelet therapy (9) have been brought up and holds promising in treating adenomyosis. Unraveling the therapeutic mechanism of those novel drugs is needed in the future. An in-depth investigation of the etiology and pathogenesis of adenomyosis could also accelerate novel drug development.

Despite several hypotheses proposed so far including microtrauma of the junctional zone, metaplasia of stem cells in the myometrium, and invagination of endometrial tissue into the myometrium, the precise pathogenesis of adenomyosis remains unknown (10, 11). Previously, common features were discovered including enhanced proliferation and high invasive capacity of endometrial cells derived from women with adenomyosis (12, 13). A recent study utilizing single-cell transcriptomic technology revealed that enhanced endometrial cell proliferation, migration, and resistance to apoptosis contribute to the invasion of the endometrium into the myometrium in women with adenomyosis (14). Moreover, an increasing number of molecular studies have reported abnormal activation of canonical pathways in the endometrium of women with adenomyosis such as eukaryotic initiation of factor-2 (EIF2) signaling, oxidative phosphorylation, mTOR, IL-6, ERK/MAPK, and TGF-beta signaling as well as altered expression of estrogen and progesterone receptors (15–18). Studies have also reported that dysregulation of long non-coding RNAs and microRNAs may contribute to the aberrant gene functions observed in the endometrial and myometrial compartments of adenomyosis patients (15, 16).

It has been proposed that increased levels of prolactin (PRL) may contribute to the development of adenomyosis. PRL is mainly produced and secreted by the pituitary gland but also in small amounts in the human endometrium and myometrium and the decidua during pregnancy (17, 18). In murine uteri, minimal elevation of serum PRL was sufficient to cause adenomyosis (19, 20). Several animal models have shown increased uterine concentrations of PRL to be a risk factor for adenomyosis (21, 22).

Bromocriptine is a dopamine receptor agonist that is commonly used to treat hyperprolactinemia by activating dopamine D2 receptors and augmenting low hypothalamic dopamine secretions, therefore restraining high PRL levels through a negative feedback loop (23, 24). Our previous clinical trial showed that vaginal bromocriptine treatment for six months significantly improves symptoms of patients with diffuse adenomyosis including menstrual bleeding and pelvic pain (7, 8) and resulted in imaging characteristics changes following treatment (7). However, the underlying mechanism of bromocriptine in reducing adenomyosis-associated symptoms remains unknown.

The objective of the study was to investigate the effect of bromocriptine on endometrial tissue from women with adenomyosis *in vivo* and primary endometrial stromal cells from the same women *in vitro*. To achieve this, we tested the expression of a proliferation marker and prolactin on endometrial tissue before and after bromocriptine treatment, cultured primary endometrial stromal cells from women with adenomyosis, and assessed their proliferation and migration ability while exposing the cells to bromocriptine treatment *in vitro*. To further explore the molecular mechanism of action of bromocriptine, small RNA sequencing was conducted on endometrial tissue to identify differentially expressed microRNAs. Predicted target genes of differentially expressed microRNAs were further analyzed using KEGG functional pathway enrichment.

## Materials and methods

### Specimen collection

The study was approved by the regional ethics board at Karolinska Institutet, Stockholm, Sweden (2013/2060-31/1) and registered at Eudract.ema.europa.eu (EudraCT 2013-004409-14). Informed consent was given to and signed by the study participants before commencing any study-related activity. In total, six patients diagnosed with diffuse adenomyosis without other known gynecological disorders such as endometriosis or myomas were recruited in this study. All patients had regular menstrual cycles. A daily dose of 5 mg bromocriptine was provided to patients *via* the vaginal route of administration for six months, as described in the clinical trial (8). Diffuse adenomyosis was clinically diagnosed based on symptoms and transvaginal sonography and magnetic resonance imaging. All the endometrial biopsies from women with adenomyosis before and after bromocriptine treatment were collected during the proliferative phase according to the last menstrual period. The collected fresh specimens were immediately fixed, frozen, and isolated for the following experiments.

### Immunohistochemistry staining

Formalin-fixed and paraffin-embedded specimens were used for immunohistochemistry staining. Six baseline samples and four after-treatment samples were included. Fewer after-treatment samples were included due to sample loss. For each sample, three replicates were prepared. 5 μm sections of paraffin-embedded endometrial tissues were prepared and immunohistochemistry staining was performed using a standardized protocol (25). Primary antibody against Ki67 (catalog no. NB110-89717, Novus biologicals, Biotechne, USA) were diluted in 1:200 using diluent DaVinci Green (Biocare Medical, Concord, CA) and incubated overnight at 4 °C. Rabbit MACH 3TM Probe and its respective HRP polymer (Biocare Medical, Concord, CA) and Betazoid DAB Chromogen (Biocare Medical, Concord, CA) were used to detect the antibody. Finally, tissue sections were counterstained using hematoxylin (Vector Laboratories, Inc., Burlingame, CA) and mounted using the xylene-based medium Pertex^®^ (Histolab, Gothenburg, Sweden). Immunopositivity for Ki67 stained areas (six randomly selected areas per slide) was analyzed at 20x magnification using Image J software.

### Endometrial stromal cell isolation and identification

Primary endometrial stromal cells from women with adenomyosis were isolated according to a protocol with minor modifications (25). Briefly, endometrial tissues were homogenized and treated in sequential steps of pancreatin-0.05% trypsin enzymatic solution, collagenase 4 (0.1 U/ml), and DNase I (16 μg/ml) solution in Ca2+/Mg2+-free PBS (Gibco^®^ Thermo Fisher Scientific, Sweden) and incubated for 30 minutes at each step. Enzymatically digested cell suspensions were filtered through a 100 µM cell strainer to remove larger debris while collecting the flowthrough containing both the epithelial and stromal fractions. Later, these cell suspensions were adherent and expanded *in vitro* for two-three generations, frozen, and stored in liquid nitrogen for the following experiments. The isolated ESC was cultured in Dulbecco’s modified Eagle’s medium (DMEM)/F-12 (Gibco, USA) containing 10% fetal bovine serum (Gibco, USA) and 1% Penicillin-Streptomycin solutions (Gibco, USA). When the cells reached 80-90% confluency, the cells were passaged, and the medium was changed every 2-3 days. Cyto-immunofluorescent staining for vimentin (Abcam, ab16700) and pan-cytokeratin (Abcam, ab86734) was applied for confirmation of stromal cell phenotype.

### Cell viability assay

Cell viability assay was assessed on ESC at baseline for determining the optimal half-maximum inhibitory concentration (IC50) under the treatment of bromocriptine (2-bromo-a-ergocryptine methane sulfonate salt). Bromocriptine was purchased from Sigma-Aldrich (Sweden, AB). 7.5mg bromocriptine powder was dissolved in 1 ml of absolute ethanol to get a 10 mM as stock concentration. Cell complete medium was used to prepare a bromocriptine working solution. CellTiter-Blue^®^ Cell Viability kit (Catalog no.G8081, Promega Biotech AB, Stockholm, Sweden) was used to detect the fluorescence intensity after 48 hours of bromocriptine treatment. Bromocriptine dissolved in ethanol was added to the culture medium of controlled cells. The kit applies a fluorescence-based method by detecting the reduction of the indicator dye, resazurin. The fluorescent signal from the CellTiter-Blue^®^ Reagent is proportional to the number of viable cells. Briefly, cells were seeded in 96-well culture plates at a concentration of 5000 cells/100ul per well. After cell adherence, various concentrations of bromocriptine (5, 10, 20, 40, 80, 160, 320 µM) were added accordingly in a 96-well plate for 48 hours. CellTiter-Blue^®^ reagents were added 4 hours before the end of treatment. A 96-well filter-based multi-mode microplate reader FLUOstar Omega (BMG LABTECH, Ortenberg, Germany) was used to detect fluorescence intensity at 590 nm, normalized to the respective vehicle treatment. The drug dose-response inhibitory curve was plotted by GraphPad 9.0 to capture the IC50 value.

### Cell proliferation assay

The proliferative ability of ESC after treatment with bromocriptine was assessed using the BrdU cell proliferation ELISA kit (catalog no. ab126556; Abcam) in accordance with the manufacturer’s protocol. Briefly, cells were seeded in a 96-well plate at a concentration of 5000 cells/well and incubated for 24 hours. Cells were then treated with bromocriptine for 48 hours. 20μL BrdU was added at least 2 hours before the termination of treatment. Next, cells were fixed for 30 minutes and incubated with the human anti-BrdU antibody for 1 hour at room temperature. After washing with washing buffer three times, 100 ul peroxidase goat anti-mouse IgG was added to each well and incubated for 30 minutes. In the end, absorbance was measured at 450 nm wavelength by a microplate reader FLUOstar Omega (BMG LABTECH, Ortenberg, Germany).

### Migration assay

To test the impact of bromocriptine on cell migration *in vitro*, a wound healing assay, and transwell migration assay was utilized to evaluate the horizontal and vertical migratory ability respectively. For the wound healing assay, cells were seeded in Culture-Insert 2 Well in a µ-Dish35mm, high (ibidi, 80206, Germany). After attachment, the inserts were gently removed to create gaps, and cells were treated with or without bromocriptine. Images were taken at different time points after insert removal (0, 12, 24, 36h). The percentage of the area covered by migrated cells was calculated by ImageJ software. For the transwell migration assay, 2×104 cells were seeded on 8.0 µm transwell migration (catalog no.353097, Corning) in a 24-well plate (catalog no.353504, Corning) containing a concentration of 2% fetal bovine serum (FBS) in DMEMF12 medium. The lower chamber contained 10% FBS as a chemoattractant. After 12, 24, and 36 hours, inserts were taken out and fixed with methanol, and washed with PBS. The migrated cells on the bottom of the inserts were stained with crystal violet. The non-migrated cells on top of the inserts were removed by gently wiping them with a cotton swab. The number of stained cells was counted in four representative areas in each well under 4X magnification using a light microscope (Nikon TS100).

### Real-time PCR

Total RNA was extracted from the bromocriptine-treated ESC as well as frozen tissues at baseline and after bromocriptine treatment using a Zymo Quick-RNA microprep kit (Zymo, USA) according to manufacturers’ protocol. The cell RNA was only extracted from three samples due to the remaining three samples were of higher passage. The eluted RNA was used immediately for cDNA conversion. cDNA was generated using SuperScript^®^ VILO™ kit (Invitrogen^®^, Thermo Fisher Scientific, Waltham, USA). Shortly, the protocol required the preparation of an RNA-free master mix which was added to 0.2 ml PCR tubes along with RNA from each sample. The following thermocycling conditions were used to generate cDNA: 10 minutes at 25 degrees, 60 minutes at 42 degrees, and finally 5 minutes at 85 degrees. The Taqman^®^ gene probe for Ki67 (Hs01032443_m1) was used for real-time PCR. The real-time PCR reaction condition consists of 20 seconds of holding stage at 95 degrees and 40 cycles of a one-second denaturation at 95 degrees and then 20 seconds of annealing and extension at 60 degrees. Gene expressions were performed and quantified by a StepOne Plus Real-time PCR instrument (Applied Biosystems, Foster City, CA, USA). Experiments were performed in triplicates. Ribosomal RNA 18s (4319413E) was used as a housekeeping gene to normalize the expression of the target gene. Fold change was calculated using the comparative Ct method.

### Enzyme-linked immunosorbent assay

Protein lysates were extracted from endometrial tissues collected at baseline and bromocriptine treatment using Pierce ^®^ RIPA lysis and extraction buffer (catalog no. 89900; Thermo Fisher Scientific, Waltham, USA) supplemented with complete™, Mini, EDTA-free protease inhibitor cocktail (catalog no. 4693159001; Sigma-Aldrich, USA). Total protein was quantified using a Qubit protein assay kit. All samples were diluted to 50 ng/ml total protein. PRL levels were then measured using the Prolactin ELISA kit (catalog no. ab108679; Abcam, UK), according to the manufacturer’s protocol. Briefly, this kit uses a competitive ELISA method wherein, the plate is pre-coated with capture antibody. Tissue lysate from endometrial samples and standards provided in the kit were dispensed as duplicates to each well along with Prolactin-HRP conjugates followed by 60 minutes of incubation. Later, they were detected using a TMB-substrate solution by measuring the absorbance at 450 nm. On the cellular level, the conditioned medium of cells was collected after 48 hours of treatment with 113.3 µM bromocriptine and centrifuged at 12000 rpm for 5 minutes at 4°C to remove cell debris. The supernatant was then stored in a -80°C freezer for the following assays. A Prolactin ELISA kit (catalog no. EHIAPRL, Invitrogen) was used to quantify the PRL level in the conditioned medium. Briefly, the standards were diluted accordingly, and samples were diluted 50-fold from the original conditioned medium. 50 µl diluted medium was added per well. After adding the stop solution, the absorbance value was measured by a microplate reader FLUOstar Omega (BMG LABTECH, Ortenberg, Germany) at the wavelength of 450nm.

### Small RNA sequencing

Library preparation: Total RNA was extracted from tissue stored in RNA later using RNeasy total RNA Kit (Qiagen, Hilden, Germany) as per the manufacturer’s protocol. cDNA libraries for small RNA sequencing were constructed according to a highly sensitive small RNA sequencing protocol (26) with 1ng of total RNA as starting material. Each sample was indexed using customized barcodes (IDT Technologies, Germany), and the 10ng library from each is pooled and sequenced. A thermal cycler from BIOER Life Touch (Techtum, China) was used in all the steps. The final cDNA libraries quality was checked on a High Sensitivity DNA chip (Agilent Technologies, USA) using Agilent 2100 Bioanalyzer System (Agilent Technologies, USA). DNA quantity was determined with Qubit Flex Fluorometer (Invitrogen, Singapore) using the Qubit 1X dsDNA High Sensitivity Kit (Invitrogen, Oregon, USA). Sequencing was performed on the Illumina NextSeq 550 platform with 1x75 basepairs, and single-end reads at the Bioinformatics and Expression Analysis core facility at Karolinska University Hospital, Sweden.

### Bioinformatics analysis

The bioinformatics analysis pipeline of small RNA sequencing is mainly adopted from a previously published pipeline with minor modifications (27). Briefly, quality control of the FASTQ files was done using the FastQC (v0.11.9) software. In the following preprocessing steps, UMI sequences were removed and appended to the read’s header for later UMI analysis. Reads were further trimmed for Illumina 3’adapter and the two cytosine-adenine (CA) bases linked to the UMI. The trimmed reads were aligned to the hg38 genome using STAR (version 2.7.2a). A read length filter of maximal 40nt was subsequently applied to the aligned reads (28). PCR duplicates were removed to create absolute molecule counts, and precursor molecule reads were filtered (27) and annotated using Mirbase (miRNAs) and GtRNAdb (tRNAs). The Bioconductor software DESeq (version 1.24.0) was used to identify differentially expressed miRNAs between the groups. Differentially expressed miRNAs with a false discovery rate (FDR) < 0.05 and a fold change (FC) of < -2 or > 2 were considered biologically and statistically significant. Raw data files are deposited in NCBI’s Gene Expression Omnibus and are accessible using the GEO Series accession number: GSE207522. Experimentally validated target genes for selected miRNAs of interest were identified using MiRTarBase (Release 8.0) (26). Target genes with both strong and weak experimental evidence were included in the downstream analysis. Biological pathways enriched among the miRNA target genes were analyzed using the web tool g:Profiler (version e101_eg48_p14_baf17f0) (29) by utilizing the default g:SCS algorithm. An adjusted p-value of <0.05 was considered statistically significant.

### Statistical analysis

GraphPad Prism 9 (GraphPad Software Inc., USA) was used for statistical analysis and graphical illustrations. The continuous variables were described as the mean ± standard deviation (SD). Two-sided Mann-Whitney Test was performed to test the Ki67 protein and gene level difference, and BrdU proliferation level difference. Two-sided Wilcoxon matched-pairs signed rank test was performed to test the tissue PRL level. IC50 was obtained by generating a dose-response curve. Two-sided paired t-test was performed to determine the PRL level in the conditioned medium. P<0.05 was considered statistically significant.

## Results

### Bromocriptine exhibits an anti-proliferative effect on endometrium from women with adenomyosis

**Figure 1:**
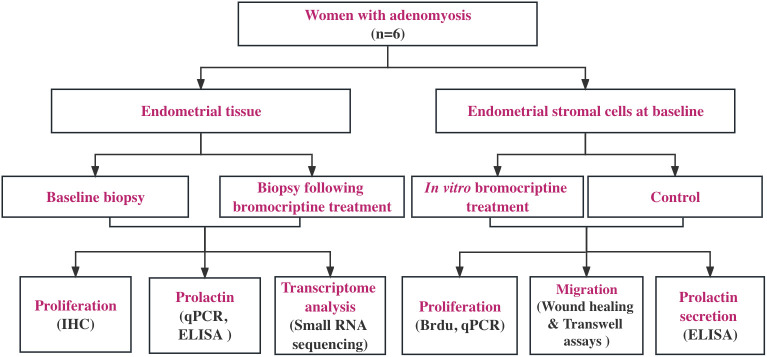
Study flow chart. Six women with diffuse adenomyosis who were previously enrolled in our pilot study (Andersson et al., 2019) were enrolled for the assessment of cellular and molecular changes induced by vaginal bromocriptine treatment. All the endometrial biopsies were collected during the proliferative phase according to the last menstrual period. The biological action of the bromocriptine was explored *in vivo* and *in vitro* separately. Proliferation analysis on tissues at baseline and after treatment was performed using immunohistochemistry (IHC). Local prolactin concentration and mRNA expression level were measured by ELISA and real-time PCR. Small RNA sequencing was performed, and the data were analyzed using bioinformatic software (Partek, R) to understand bromocriptine-induced changes on the transcriptomic level. For *in vitro* experiment, after determining the optimal half-maximal inhibitory concentration of bromocriptine, cell proliferation, and migration assays were used to evaluate the effect of bromocriptine on the cultured cells. The prolactin level in the conditioned medium of cells was measured by ELISA.

**Figure 2:**
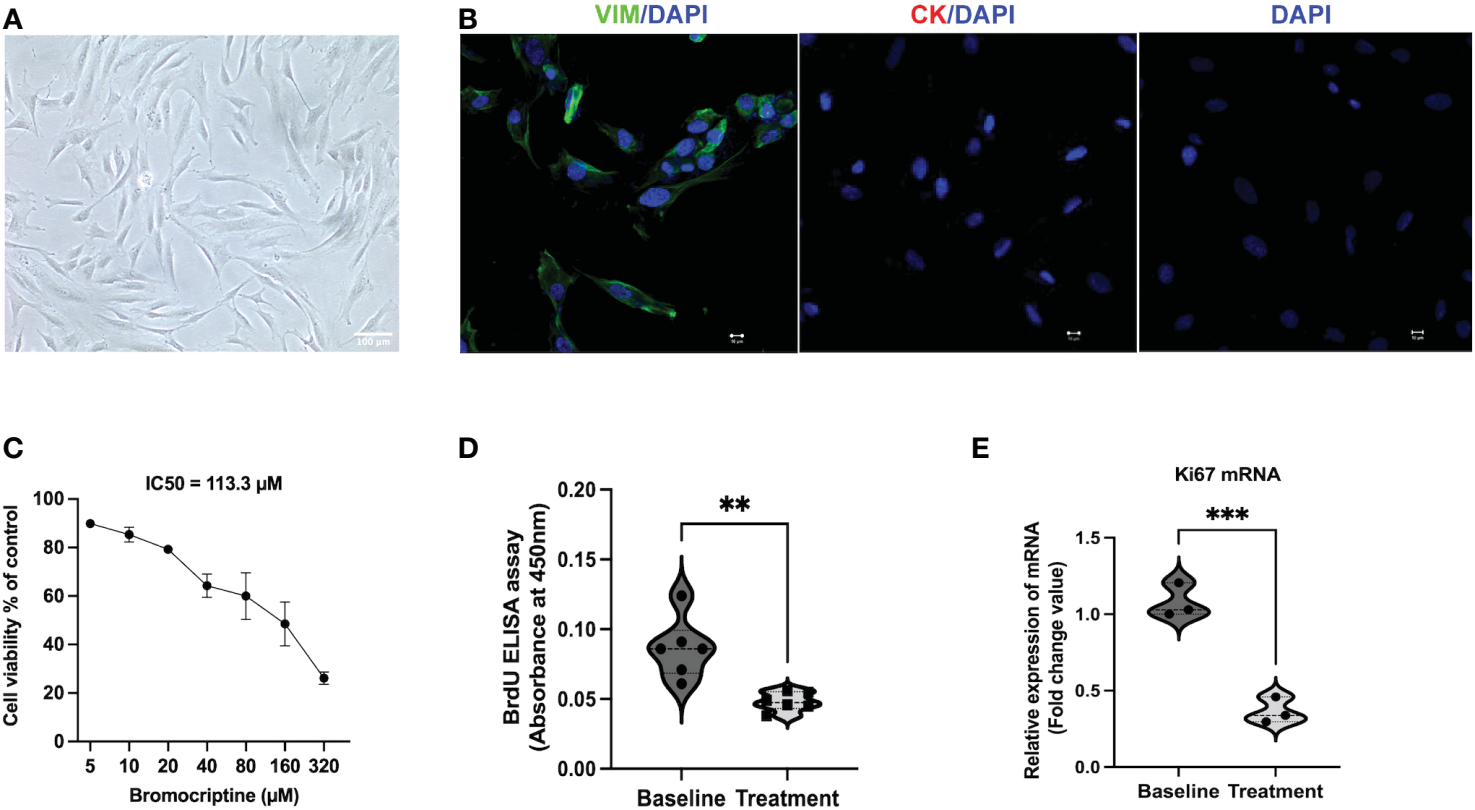
Identification of endometrial stromal cells derived from women with adenomyosis and the effect of bromocriptine on cell proliferative ability *in vitro*. **(A)** The primary endometrial stromal cells in adenomyosis showed a typical spindle-shaped morphology under a light microscope (Magnification of 20×, scale bar:100 μm). **(B)** Cultured cells were positive for vimentin (Green) and negative for cytokeratin (Red) by immunofluorescent staining. **(C)** Effect of various doses of bromocriptine (5, 10, 20, 40, 80, 160, 320 μM) on endometrial stromal cells (n=3). After 48 hours treatment of with bromocriptine, the viability of the cells decreased dose-dependently with an IC50 value of 113.3 μM. **(D)** Bromocriptine inhibited the proliferation of endometrial stromal cells by BrdU proliferation assay. **(E)** Effect of 113.3 μM bromocriptine treatment for 48 hours on Ki67 mRNA expression in cultured ADS-ESCs. 18sRNA was used as a reference gene. The analysis and plot were generated by GraphPad 9.0. **P < 0.01 and ***P < 0.001 indicate significant differences.

**Figure 3:**
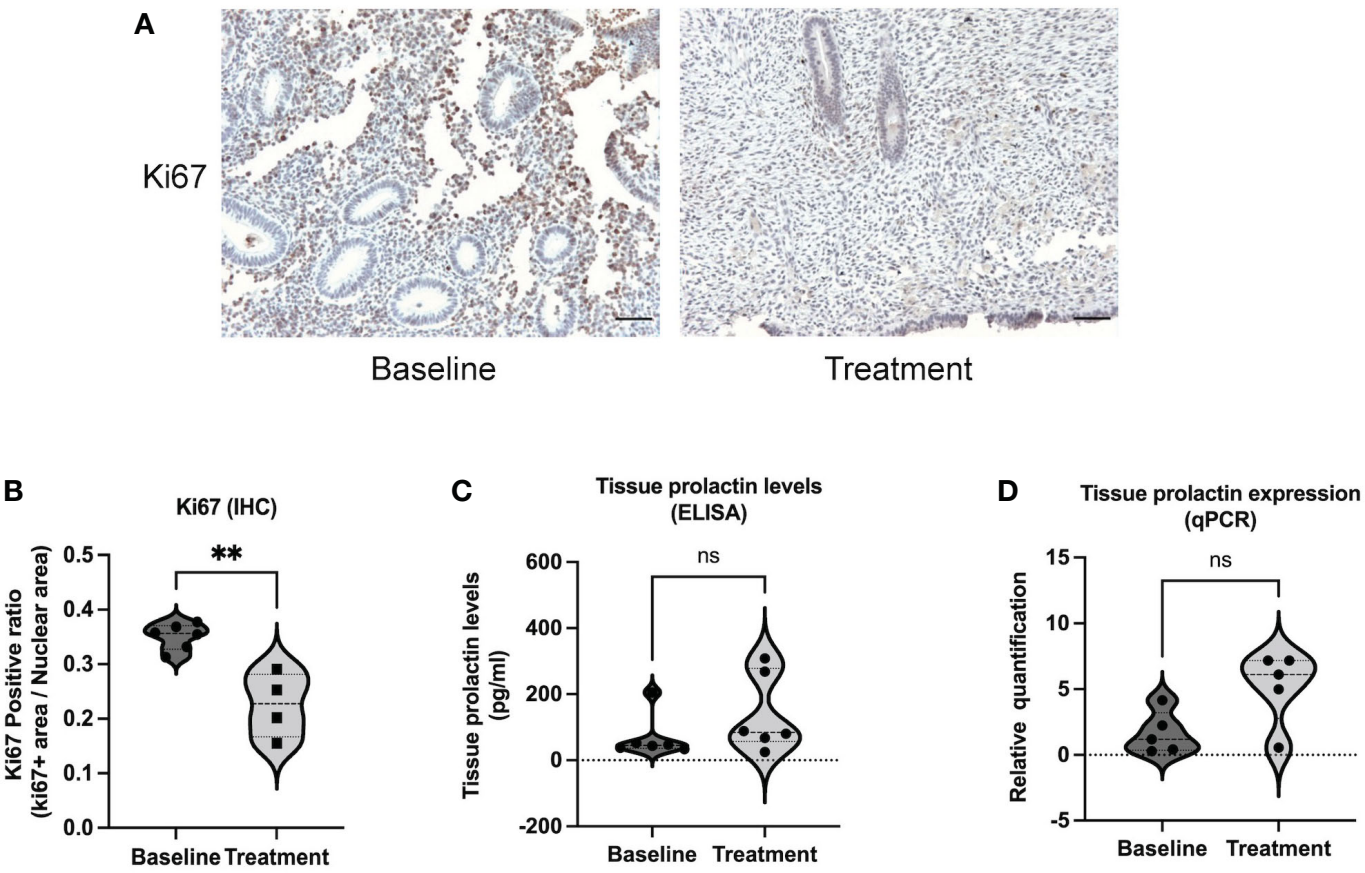
Ki67 and prolactin expression in endometrial tissue between baseline and bromocriptine treatment in women with adenomyosis. **(A)** Representative images of Ki67 immunohistochemistry staining at baseline and after treatment (Magnification of 20×, scale bar:100 μm). **(B)** The Ki67 positive ratio in endometrial tissue was quantified by ImageJ. The protein levels of Ki67 decreased significantly after the treatment of bromocriptine (n=6 for the baseline and n=4 for the treatment). **(C)** Prolactin concentration in endometrial tissue was evaluated by ELISA (n=6 both for the baseline and treatment). **(D)** Tissue prolactin mRNA expression levels were determined using real-time PCR (n=5 both for the baseline and treatment). The analysis and plot were generated by GraphPad 9.0. Two-tailed Mann-Whitney U test was performed. **P < 0.01 indicates a significant difference. ns indicates no significant difference.

As shown in the study flow chart (**Figure 1**), we determined the expression level of the proliferative marker, Ki67 in the endometrial tissue from baseline and after bromocriptine treatment. To further investigate the anti-proliferative effect of bromocriptine. The primary endometrial stromal cells (**Figure 2A**) of women with adenomyosis were isolated and identified by vimentin and cytokeratin immunofluorescent staining (**Figure 2B**). Cells were treated with different doses of bromocriptine (5, 10, 20, 40, 80, 160, 320 μM) for 48 hours, and cell viability decreased in a dose-dependent manner, showing a cytotoxic effect. The half-maximal inhibitory concentration (IC50) value of bromocriptine towards ESC was 113.3 μM (**Figure 2C**). The results showed a significant reduction of Ki67 protein expression in endometrial tissue from women with adenomyosis after bromocriptine treatment (**Figures 3A, B**). Furthermore, the BrdU ELISA assay showed that the proliferative ability of ESC decreased significantly after treatment with a 113.3 μM dose of bromocriptine for 48 hours (**Figure 2D**). Ki67 gene expression was also reduced in the ESC treated with bromocriptine *in vitro* (**Figure 2E**). In summary, we found that bromocriptine could inhibit endometrial proliferation in adenomyosis both *in vivo* and *in vitro*.

### Bromocriptine reduces the migratory ability of endometrial stromal cells in adenomyosis

**Figure 4:**
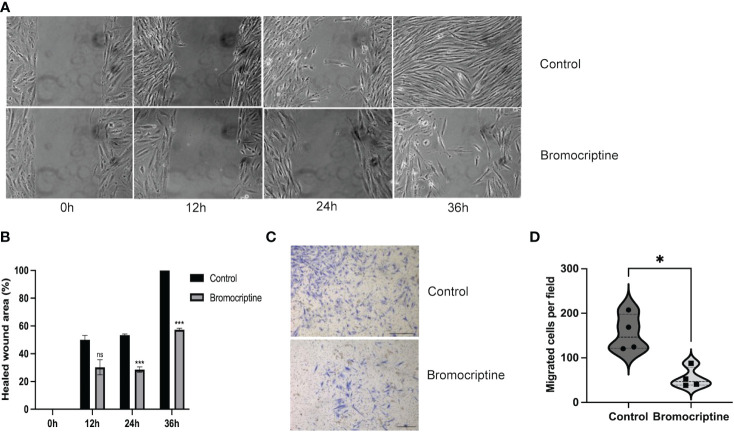
Effect of bromocriptine on cell migration ability. **(A)** Representative bright-field images of the wound healing area at 0h, 12h, 24h, and 36h (Magnification of 20×). **(B)** Statistical analysis of healed wound area in bromocriptine and control groups. A dose of 113.3 μM bromocriptine treatment significantly inhibited the cell’s horizontal migratory ability at 24h and 36h. **(C)** Representative images of the bottom surface of a transwell migration assay in endometrial stromal cells (Magnification of 4×, scale bar:500 μm). **(D)** Quantification of migrated cells. The number of migrated cells in the bromocriptine group reduced significantly compared to the control group. The analysis and plot were generated by GraphPad 9.0. *P < 0.05, ***P < 0.001, ns indicates no significant difference.

It has been reported that the high invasiveness of adenomyosis endometrium may be involved in the development of adenomyosis (12). To explore the effect of bromocriptine on the migratory ability of ESC, the cells were cultured in DMEM/F12 medium with 2% fetal bovine serum and 113.3 μM bromocriptine. DMEM/F12 with 2% fetal bovine serum was used as the control group. After 24 hours and 36 hours of treatment, the horizontal migratory ability of cells was significantly decreased in the 113.3 μM bromocriptine group compared with the control group (**Figure 4A, B**). For the transwell migration assay, DMEM/F12 medium with 10% fetal bovine serum was added to the lower chamber as an attractant to induce cell migration. In line with the above finding, the vertical migratory ability of adenomyotic ESC was also significantly lower in the bromocriptine group compared to the control group (**Figure 4C, D**). So, bromocriptine inhibits the migratory ability of endometrial stromal cells in adenomyosis *in vitro*.

### Local prolactin level does not correlate with the clinical effects of bromocriptine

Several animal studies have shown that uterine PRL may contribute to the pathogenesis of adenomyosis (21, 22). To study the influence of bromocriptine on local PRL secretion, protein and RNA were extracted from the endometrial tissue of women with adenomyosis before and after bromocriptine treatment. No significant difference was observed in either PRL mRNA expression level or concentrations of PRL in endometrial tissue after bromocriptine treatment (**Figures 3C, D**). To explore whether bromocriptine affects the PRL secretion of adenomyotic ESC *in vitro*, PRL levels in a conditioned medium of ESC were measured after treatment with bromocriptine for 48 hours by ELISA. In line with the *in-vivo* data, no significant difference was identified between the bromocriptine-treated and untreated control groups (**Figure S1**). These results indicate that the effect of bromocriptine in treating adenomyosis-related symptoms may not correlate with local PRL levels in endometrial tissue from women with adenomyosis.

### Upregulated microRNAs after bromocriptine treatment are predicted to affect proliferation-associated pathways

**Figure 5:**
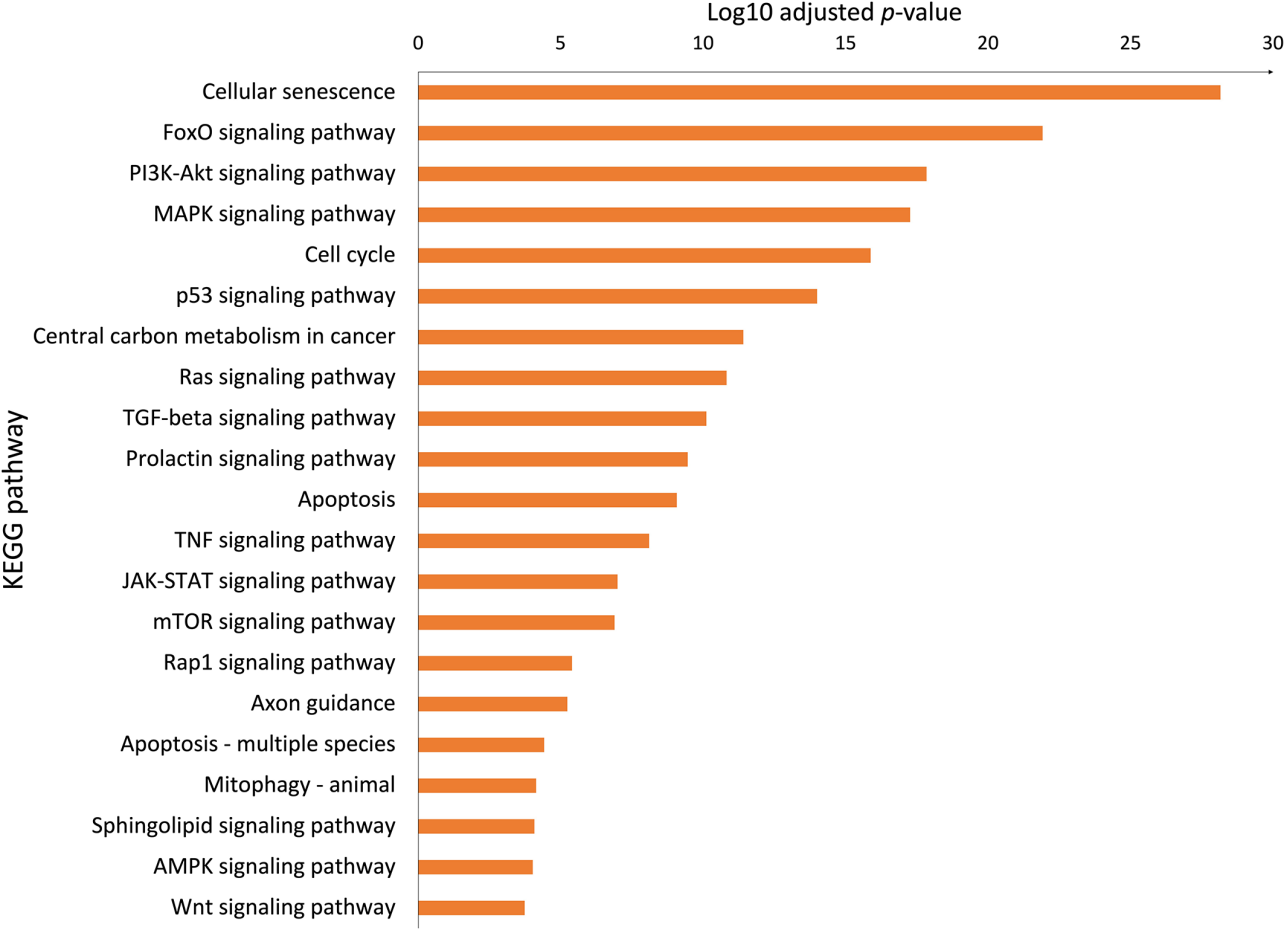
Biological pathways associated with cell proliferation and apoptosis. The Bar chart showed significant KEGG pathways involved in cell proliferation and apoptosis enriched the upregulated microRNAs (adjusted P-value < 0.05, selected from 122 pathways).

To better understand the molecular mechanism of bromocriptine in adenomyosis, small RNA sequencing was conducted on endometrium from women with adenomyosis before and after six months of vaginal bromocriptine treatment. In total, 27 miRNAs (-2 < FC < 2; *P*-value <0.05) were significantly upregulated after bromocriptine treatment (**Table 1**). To explore how the differentially expressed miRNAs may be involved in the treatment effect of bromocriptine, we predicted target genes of the differentially expressed miRNAs followed by KEGG pathway enrichment analysis. In total, 27 miRNAs were predicted to target 572 genes. Pathway enrichment analysis further showed that the predicted target genes of the miRNAs were significantly enriched in 122 KEGG pathways. Out of these 122 pathways, 21 signaling pathways were associated with cell proliferation and apoptosis (**Figure 5**).

**Table 1:** 27 upregulated differentially expressed microRNAs in women with adenomyosis after bromocriptine treatment compared to before bromocriptine treatment.

Differentially expressed microRNAs (Treatment Vs Baseline)	Mean counts (Baseline, B)	Mean counts (Treatment, T)	Fold change (T Vs B)	Adjusted P value (T Vs B)
hsa-let-7a-3p	2	21	7,42	0,01543418
hsa-miR-210-3p	3	25	5,94	0,03466447
hsa-miR-486-3p	2	12	5,31	0,04993067
hsa-miR-106b-3p	20	135	5,04	0,01543418
hsa-miR-324-5p	1	6	4,79	0,03483399
hsa-miR-451a	54	291	4,38	0,03483399
hsa-miR-486-5p	649	3394	4,20	0,034833989
hsa-miR-497-5p	4	16	3,67	0,02510916
hsa-miR-185-5p	89	408	3,55	0,02510916
hsa-miR-1307-3p	5	20	3,31	0,049930675
hsa-miR-25-3p	112	473	3,20	0,034833989
hsa-miR-199a-3p	36	146	3,19	0,02510916
hsa-miR-3184-5p	6	23	3,11	0,034833989
hsa-miR-99b-3p	2	9	3,05	0,049930675
hsa-miR-106b-5p	3	12	3,01	0,034833989
hsa-miR-221-3p	25	96	2,92	0,034833989
hsa-miR-22-3p	101	365	2,82	0,027744514
hsa-miR-21-5p	293	1031	2,71	0,034833989
hsa-miR-425-5p	11	40	2,69	0,015462837
hsa-miR-31-5p	18	61	2,64	0,015434175
hsa-miR-30b-5p	7	23	2,50	0,034833989
hsa-miR-363-3p	50	166	2,49	0,034833989
hsa-miR-181a-5p	162	476	2,38	0,015434175
hsa-miR-361-3p	31	83	2,19	0,034833989
hsa-miR-103b	62	165	2,17	0,034664469
hsa-miR-30e-5p	228	650	2,16	0,049930675
hsa-let-7d-3p	14	36	2,10	0,034833989

## Discussion

In the present study, we investigated the cellular and molecular changes of the endometrium after bromocriptine treatment in women with adenomyosis. Our results revealed that bromocriptine inhibits the proliferation of endometrial cells in women with adenomyosis both *in vitro* and *in vivo*. Furthermore, bromocriptine also reduces the migratory ability of ESC *in vitro*. In line with these results, small RNA sequencing data further showed that bromocriptine treatment upregulated the expression of several miRNAs which were predicted to affect biological pathways associated with cell proliferation. Overall, the findings of this study lay the foundation for further exploration of the potential clinical utility of bromocriptine as an effective treatment for adenomyosis.

Common features of adenomyosis include the increased capacity of endometrial cells to proliferate, migrate and invade the myometrium (11). Several studies suggested that the enhanced proliferative and migratory properties of endometrial cells derived from adenomyosis may contribute to the infiltration of endometrial cells in the myometrium as a critical step in disease development (30–32). The cell proliferation marker, Ki67 was significantly decreased after bromocriptine treatment *in vivo*. Moreover, we found that bromocriptine inhibited the proliferation of ESC cultured *in vitro* in a dose-dependent way. 113.3 µM was found to be the optimal dosage for bromocriptine to exert inhibitory effects. Future *in vivo* studies for the selection of the optimal dose of bromocriptine for future clinical use is required. In addition to cell proliferation, enhanced cell migratory ability could also contribute to the development of adenomyosis. Hence, the effect of bromocriptine on the migratory ability of stromal cells was evaluated by scratch and transwell migration assays. Our results suggest that bromocriptine decreases the proliferation and migration potential of endometrial cells in adenomyosis.

MicroRNAs are a group of small nucleotides that usually act as gene silencers. They play important roles in regulating gene expression, exerting many biological actions such as anti-proliferative, anti-migrative, and pro-apoptotic effects. Adenomyosis has been linked to changes in the miRNA profile of the endometrium (16). In the above study, several differentially expressed miRNAs expressed in the endometrium of adenomyosis patients have been suggested as diagnostic markers with relatively high sensitivity and specificity. In our study, we speculate that bromocriptine exerts an anti-cell proliferation action by silencing specific gene expression by activating several miRNAs expression. Sheu et al. reported that upregulation of hsa-miR-486-3p results in the downregulation of four genes (DDX11, E2F1, NPTX1, and PLXNA4), which may contribute to decreased proliferation of fibroblasts from idiopathic pulmonary fibrosis (33). In line with the above study, our study showed that hsa-miR-486-3p was one of the top three upregulated miRNAs in the endometrium which may be involved in the decreased cell proliferation after the bromocriptine treatment. Another study indicated that 15 upregulated miRNAs in senescent human fibroblast cells may act as promoters of cell cycle arrest and telomere degradation, resulting in a senescent phenotype in the end (34). Among the above 15 miRNAs, hsa-miR-181a-5p and hsa-miR-221-3p overlapped with our results. The two overlapping miRNAs may reflect the possible role of cellular senescence in the bromocriptine-induced changes in the endometrium of women with adenomyosis. This was further supported by the biological pathway analysis which showed that cellular senescence was among the most significantly enriched pathways. Previously, studies have reported that aberrant activation of key growth signaling pathways such as PI3K-AKT signaling (15) and ERK/MAPK signaling (30) were present in the endometrium of women with adenomyosis. In line with the above reports, we found that PI3K-AKT, Cell cycle, JAK-STAT, and several signaling pathways associated with cell proliferation are involved in the bromocriptine effects on adenomyosis-related symptoms. Besides the proliferation pathways, we do see several KEGG pathways associated with cell migratory function such as TGF-beta, and Wnt signaling pathways. Those signaling pathways are not at the top of the KEGG ranking. The key regulators may intertwine with proliferation pathways and might involve many cell biology functions and therefore may facilitate the invasion of stromal cells to the myometrium.

As a key reproductive hormone, PRL contributes to many physiological functions in maintaining women’s health such as breast development and lactation. Except for the principal site of PRL production, the anterior pituitary gland, several new potential sites have been found such as endometrial and myometrial tissue (35, 36). PRL is synthesized in the human endometrium during the late luteal phase and pregnancy. Progesterone stimulates PRL secretion and its receptor expression when endometrial stromal cells differentiate into decidual cells (37, 38). However, PRL local secretion pattern in healthy human endometrium during the remaining menstrual cycle is unclear so far. It is reported that uterine prolactin has the same protein structure as pituitary prolactin but does have a different 5’ regulatory region (18). The prolactin receptor is present in the uterus and uterine prolactin also appears to act as a local growth factor and a smooth muscle cell mitogen (39). The tissue-specific secretion of PRL may also be influenced by serum PRL circulation, other hormones, and pathological conditions. As far as we know, the current evidence for the prolactin hypothesis was promoted by murine models of adenomyosis (19, 40). Subsequent studies suggested there may be evidence for the role of prolactin in human adenomyosis (41). To test the prolactin hypothesis, we compared the local tissue PRL level before and after bromocriptine treatment. However, we did not observe a significant reduction in PRL level in the endometrium in women with adenomyosis after bromocriptine treatment. Furthermore, the PRL level in the conditioned medium of bromocriptine-treated stromal cells remained unchanged as well. These negative results might indicate that bromocriptine may not be involved in local PRL regulation in endometrial tissue. However, a small sample size can compromise the conclusions drawn from the study. Though PRL receptor was discovered both in the myometrium and endometrium indicating a possible role of PRL in the baboon uterus (40), the action-targeted locations and cell types within the uterus induced by PRL remain unclear. As far as we know, the current evidence for the prolactin hypothesis is mainly derived from animal studies. Hence, considering the complex pathologies of adenomyosis, more well-designed human studies including healthy controls are needed to elucidate PRL expression patterns in endometrial tissue, as well as the role of dopamine in PRL regulation in endometrial tissue in humans. Based on the current evidence from this study, we could conclude that the local endometrial PRL level does not correlate with the anti-proliferation effect of bromocriptine in adenomyosis.

In our previous pilot trial, we chose the vaginal route to obtain a high local uterine concentration of bromocriptine and to minimize side effects. Six months duration allowed the bromocriptine to fully exert its function, reflected in the improvement of symptoms. In the follow-up study now, the exploration of the *in vivo* and *in vitro* effects of bromocriptine treatment on adenomyosis shows that vaginal bromocriptine improved symptoms in adenomyosis could potentially be explained by inhibiting the proliferation of endometrial cells. However, there are several limitations of our study including the small sample size and lack of primary epithelial cells. The pathogenesis of adenomyosis is unclear, so far there is no single hypothesis that could explain all pathological alterations. Currently, many studies do observe higher proliferative and migratory properties of the endometrium of women diagnosed with adenomyosis. However, whether such endometrial changes are the cause or consequences of adenomyosis is still debated. Besides, other factors such as inflammation, hormonal imbalance, and abnormal neoangiogenesis may also interweave with endometrial alteration during the development of adenomyosis (42).

Study limitations include small sample size, the lack of functional experiments on endometrial epithelial cells, and the lack of in-depth downstream analysis of deregulated miRNAs, which are to be addressed in future studies. Our study is a small self-controlled trial, we focused on studying the changes in pathological endometrial tissue under the influence of bromocriptine in adenomyosis. In the future, more human studies are required to elucidate the prolactin hypothesis in adenomyosis and investigate the effect of bromocriptine on healthy endometrial tissue as well.

Current treatment options are limited and invasive for adenomyosis due to the unclear pathophysiology. More drugs should be developed specifically for adenomyosis. As a pilot study, the findings of our study revealed that bromocriptine could suppress the proliferation of endometrium in women with adenomyosis both *in vitro* and *in vivo.* Moreover, several upregulated miRNAs may be involved in the antiproliferative action of bromocriptine. Further research into the underlying roles of miRNAs, signaling pathways, and molecular actions of bromocriptine in the treatment of adenomyosis are needed in the future. We consider that this mechanism may partially explain the improved symptoms observed in women with adenomyosis after bromocriptine treatment.

## Data availability statement

The datasets presented in this study can be found in online repositories. The names of the repository/repositories and accession number(s) can be found below: https://www.ncbi.nlm.nih.gov/geo/query/acc.cgi?acc=GSE207522, GSE207522.

## Ethics statement

The studies involving human participants were reviewed and approved by the regional ethics board at Karolinska Institutet, Stockholm, Sweden. The patients/participants provided their written informed consent to participate in this study

## Author contributions

ES was responsible for the clinical trial hypothesis. JA and KG-D were responsible for the clinical trial conduct including regulatory approvals. SP-S, YT, NB, and KG-D formulated the experimental study design. SP-S, YT, and CF developed the secondary study objectives and experimental approaches. JA recruited the study participants and collected the endometrial biopsies. SP-S processed clinical samples and performed preparatory steps for small RNA sequencing. YT performed *in vitro* experiments. NB and CF prepared the small RNA libraries. NB, SP-S, CF, and EK performed the bioinformatic analysis of the small RNA data. YT and CF performed functional downstream analysis and interpreted the results. YT and SP-S wrote the manuscript. SP-S, CF, ES, JA, EK, DP, PL, and KG-D critically revised the manuscript. All authors contributed to the article and approved the submitted version.

## Supplementary material

Supplementary Figure 1 Prolactin level in conditioned medium. ns indicates no significant difference between the group with adding bromocriptine and without adding bromocriptine.
