# Supplementary figures and images for: Bromocriptine inhibits proliferation in the endometrium from women with adenomyosis
Source: Front Endocrinol (Lausanne). 2023 Mar 9;14:1026168. doi: 10.3389/fendo.2023.1026168 (PMC10034369; doi:10.3389/fendo.2023.1026168)

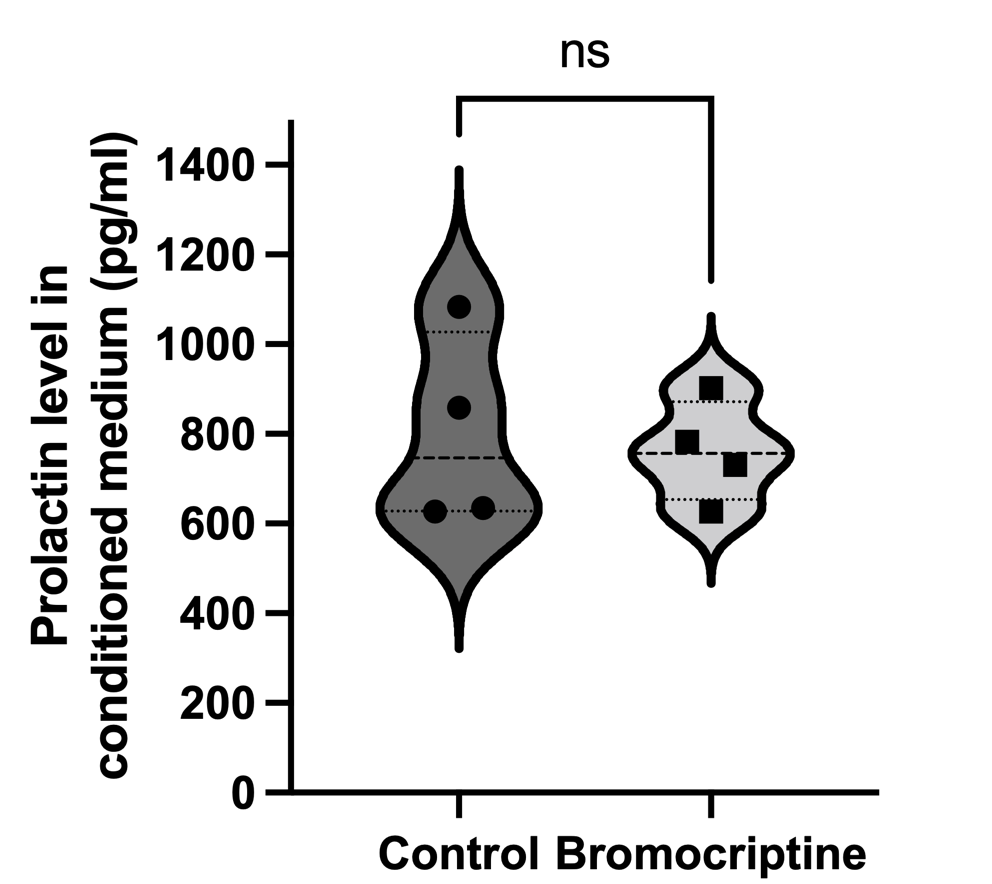

Supplement: Supplementary Figure 1 — Prolactin level in conditioned medium. ns indicates no significant difference between the group with adding bromocriptine and without adding bromocriptine. [file Image_1.tiff]
